# Population Structure of Multidrug-Resistant *Klebsiella oxytoca* within Hospitals across the United Kingdom and Ireland Identifies Sharing of Virulence and Resistance Genes with *K. pneumoniae*

**DOI:** 10.1093/gbe/evx019

**Published:** 2017-03-01

**Authors:** Danesh Moradigaravand, Veronique Martin, Sharon J. Peacock, Julian Parkhill

**Affiliations:** 1 Wellcome Trust Sanger Institute, Wellcome Genome Campus, Hinxton, Cambridgeshire, United Kingdom; 2 British Society for Antimicrobial Chemotherapy, Griffin House, Birmingham, United Kingdom; 3 Department of Medicine, University of Cambridge, Addenbrooke’s Hospital, Cambridge, United Kingdom; 4 London School of Hygiene and Tropical Medicine, London, United Kingdom

**Keywords:** microbial genomics, antimicrobial resistance, population genomics, genomic epidemiology

## Abstract

*Klebsiella oxytoca*, a member of the Enterobacteriaceae, is a gram-negative pathogenic bacterium of environmental origin, which can cause infection in healthcare settings. Outbreaks of multidrug-resistant *K. oxytoca* infection have been increasingly reported in hospitalized patients. Despite the growing importance of this pathogen, there is limited knowledge about the population structure and epidemiology of antimicrobial resistant *K. oxytoca*. We investigated the population structure and genomic basis of antimicrobial resistance of 41 multidrug resistant *K. oxytoca* isolates recovered from bloodstream infections across the UK and Ireland. Our results show that *K. oxytoca* has a highly diverse population, which is composed of several distinct clades, and we identified one recent expansion of a clone within our dataset. Although the *K. oxytoca* genomes are clearly distinct from the genomes of a global collection of *Klebsiella pneumoniae* complex, pre-dominantly composed of *K. pneumoniae*, we found evidence for sharing of core genes through recombination, as well as the exchange of accessory antimicrobial resistance and virulence factor genes between the species. Our findings also suggest that the different *K. oxytoca* clades have acquired antimicrobial resistance and virulence factor genes independently. This highlights the clinical and therapeutic importance of genetic flexibility in *K. oxytoca* and the relevance of this in its role as an opportunistic pathogen.

## Introduction

*Klebsiella oxytoca* is an important member of the genus *Klebsiella*, which contains species that cause nosocomial and community acquired infections worldwide (Bagley 1985; Broberg et al. 2014; Podschun and Ullmann 1998). Originating from the environment, *K. oxytoca* typically resides in the intestinal tract as a commensal bacterium but can spread to the bloodstream and cause disease, in particular in patients with a compromised immune system (Al-Anazi et al. 2008; Tang and Chen 1995). *Klebsiella oxytoca* outbreaks generally have environmental sources, and are subsequently propagated by the dissemination of the pathogen in healthcare settings, for example, in transplant, intensive care, or neonatal units (Bagley 1985; Decre et al. 2004; Lowe et al. 2012; Zarate et al. 2008). Furthermore, outbreaks of *K. oxytoca* usually involve strains with extended-spectrum beta-lactamases and carbapenamases (Decre et al. 2004; Lowe et al. 2012; Schulz-Stubner and Kniehl 2011; Zarate et al. 2008), which enable bacterial resistance to beta-lactam antibiotics and consequently lead to therapeutic problems (Wu et al. 1991, 1999). The mechanisms of resistance may involve either the over-expression of constitutive beta-lactamase genes or the presence of multiple copies of beta-lactamase genes, both of which result in the over-production of beta-lactamase enzymes (Gheorghiu et al. 1997; Paterson and Bonomo 2005). *Klebsiella oxytoca* has also been reported to exhibit resistance to other commonly used antibiotics, such as fluoroquinolones and tetracyclines (Brisse et al. 2000).

The genomes of several *K. oxytoca* isolates have recently been sequenced, showing that the bacterial genome is 5Mbp in length and contains 5,488 protein-coding genes (Shin et al. 2012). Some of these isolates were recovered from environmental sources (Chen et al. 2013; Lu et al. 2015) and the genomic data was generated in order to better understand their ability to produce specific chemicals, which are very useful industrial raw materials, for example, ethanol and 1,3-propanediol, in bioreactors and fuel production (Dien et al. 2003; Zhang et al. 2012). The sequencing of clinical *K. oxytoca* revealed a combination of putative virulence, antibiotic resistance and metabolic genes that provide the species with the genetic repertoire to survive and transmit within the human host and overcome specific challenges in the clinical habitat, including antibiotic treatment (Liao et al. 2012). Moreover the few previously sequenced genomes of *K. oxytoca* were shown to share some virulence genes with *K. pneumoniae* (Jiang et al. 2014), suggesting that the two closely related pathogenic species may share a common gene pool providing similar virulence and resistance mechanisms. However, due to the lack of population level data the insights that such studies on individual genomes of *K. oxytoca* can provide are limited. Furthermore, the population diversity of antimicrobial resistance and virulence genes, as well as the global population structure of *K. oxytoca*, is largely unknown. To address these, we utilized the whole genome sequencing of a nationwide systematic collection of multidrug resistant *K. oxytoca* isolated from bloodstream infections in the UK and Ireland in order to investigate the population diversity and occurrence of antimicrobial resistance determinants across the population. Our findings indicate that the *K. oxytoca* population is highly diverse and comprised of several distinct clades, one of which had evidence for recent expansion. Besides the emergence of species-specific resistance genes, our results also show similarities between *K. oxytoca* and the global *K. pneumoniae* complex population (Holt et al. 2015) and identify common virulence and antibiotic resistance mechanisms that have been acquired in one or other of the species.

## Materials and Methods

### Isolates and Antimicrobial Susceptibility Testing

Approval to conduct the study was obtained from the Research Ethics Service (ref: 12/EE/0439) of the United Kingdom and the Cambridge University Hospitals (CUH) Research and Development (R&D) Department. Forty-one *K. oxytoca* isolates were collected by the British Society for Antimicrobial Chemotherapy (BSAC) as part of a systematic bacteremia surveillance programme between 2001 and 2011 by 20 hospitals across the United Kingdom and Ireland (UK&I). A list of isolates, along with accession numbers for the genomic data, is provided in supplementary table S1, Supplementary Material online. We also analyzed the collection by placing it in the context of previously published *K. oxytoca* genomes. To conduct a comparative study between the *K. oxytoca* population and the global *K. pneumoniae* complex population, which is predominantly composed of *K. pneumoniae*, we utilized the genomes from a published global *K. pneumoniae* complex study (Holt et al. 2015).

Susceptibility to a wide range of antimicrobials was examined in this study. This included the beta-lactams; penicillins (amoxicillin, amoxicillin–clavulanate and piperacillin–tazobactam) and cephalosporins (cefuroxime, cefoxitin, cefotaxime and ceftazidime) and other antibiotics such as tetracyclines (minocycline, tetracycline and tigecycline), an aminoglycoside (gentamicin), and a fluoroquinolone (ciprofloxacin). The minimum inhibitory concentration (MIC) for each of the isolates was generated using the agar dilution method (Andrews 2001), as described in the protocol available on the BSAC surveillance programme website (www.bsacsurv.org/protocols/; MICs obtained by the BSAC agar dilution were shown to be in good agreement with the MICs from the broth microdilution method, Reynolds et al. 2003). The distributions for the antimicrobials were then compared with the distributions for those antimicrobials given on the European Committee on Antimicrobial Susceptibility Testing (EUCAST) website (the distributions of MICs for *K. oxytoca* were the same as those for *Raoultella ornithinolytica*). The EUCAST distributions, as well as the clinical breakpoints for *K. oxytoca* were downloaded from the EUCAST website on 10/01/2017.

### Sequencing and Pan-Genome Analysis

DNA was extracted with the QIAxtractor (QIAgen) kit according to the manufacturer’s instructions. Subsequently we prepared Illumina sequencing libraries with a 450 bp insert size, as detailed in the manufacturer’s protocol, and performed sequencing on an Illumina HiSeq2000 with paired-end reads of length 100 bp. We multiplexed 96 samples per lane to attain an average depth of coverage of 85-fold. An assembly improvement pipeline (Page et al. 2016) based on Velvet (Zerbino and Birney 2008) was used to obtain de novo genome assemblies. The assemblies were annotated with Prokka (Seemann 2014). The output of Prokka was used as input for the pan-genome pipeline Roary (Page et al. 2015), which produced a list of genes in the core and accessory genome. We have submitted the output files of Roary and the sequences of the genes in the pan-genome to a public repository (www.data.mendeley.com/datasets/z4n3sbszgb/1).

We identified SNPs in the core genome alignment of *K. oxytoca* using an in-house tool (www.github.com/sanger-pathogens/snp-sites). We mapped the short reads from both the global *K. pneumoniae* complex collection and our *K. oxytoca* isolates to the reference genome *K. oxytoca* KCTC (accession number: CP003218), with SMALT v0.7.4 (www.sanger.ac.uk/science/tools/smalt-0) with maximum and minimum inserts sizes of 1,000 and 50, respectively. We then employed SAMtools mpileup (Li et al. 2009) and BCFtools, as described in (Harris et al. 2010), to annotate SNPs. To this end, we considered SNPs that were supported by at least two forward and two reverse reads. We employed a minimum base call quality of 50 and a minimum root mean squared mapping quality of 30 to call a SNP. We excluded the SNPs at sites with heterogeneous mapping in which the SNP was present in <75% of reads at that site, similar to Harris et al. (2010). To construct the multiple alignment, we ignored the small indels and produced pseudo-sequences. The pseudo-sequences were then used to construct a multiple alignment of the collection. We used the same tool, as described above for the core-genome alignment, to extract SNPs from the multiple alignment.

To obtain a Maximum Likelihood tree, FastTree version 2.1.3 was used with generalized time-reversible model to calculate the phylogenetic tree from the alignments, after ignoring sites with unknown bases (Price et al. 2010). We used iTOL (Letunic and Bork 2016), FigTree (www.tree.bio.ed.ac.uk/software/figtree/) and *Dendroscope* (Huson and Scornavacca 2012) to visualize the results and compare the pattern of the presence and absence of accessory gene with the core genome content. We calculated both the pairwise SNP distances and the Average Nucleotide Identity (ANI) to measure population diversity. To calculate the ANI values, we employed the online server that is available at www.enve-omics.ce.gatech.edu/ani/ with the default parameter values and submitted the assemblies to the server. We used the ANI calculator to obtain the maximum and minimum divergences between pairs of isolates from the MDR *K. oxytoca* and each sub-species of the global *K. pneumoniae* complex collection (Holt et al. 2015) with the largest pairwise SNP distances. We also calculated the ANI values for the pairs of isolates within *K. oxytoca* and the global collection with the largest pairwise SNP distance value to obtain the maximum and minimum boundaries for the genomic diversity within each species.

### *In Silico* MLST Analysis and Identification of Antimicrobial Resistance Determinants, Virulence Factors, and Plasmids

We employed the srst2 package (Inouye et al. 2014), with the coverage cut-off of 90%, to screen the short reads for known resistance genes and plasmid replicons within the dataset. Identification of the variants of the *bla*_oxy_ gene allowed us to identify the major phylogroups of *K. oxytoca* (Fevre et al. 2005). To compare the distribution of virulence genes in the *K. oxytoca* and the global *K. pneumoniae* complex collection, we downloaded the sequences of putative virulence factors from the BIGSdb-Kp database (Bialek-Davenet et al. 2014), available on the Pasteur institute website (http://bigsdb.pasteur.fr/perl/bigsdb/bigsdb.pl?db=pubmlst_klebsiella_seqdef_public&page=downloadAlleles) and used these as reference sequences for srst2. We only used those genes which have a previously published link with virulence in *K. pneumoniae*. We identified STs of the isolates by performing in silico MLST with an in-house tool (https://github.com/sanger-pathogens/mlst_check) that takes genome assemblies.

### Phylogenetic and Recombination Analysis and Substitution Rate Calculation

After identifying the phylogenetic structure of the MDR *K. oxytoca* population, we extracted isolates within the ST2 clade and mapped the short reads to the concatenated contigs of the isolate with the best assembly statistics, that is, the highest N50 value. The mapping and SNP calling was done as described above and a multiple alignment, which includes 5,607 SNPs, was reconstructed from pseudo-genomes.

We then identified high-SNP density blocks, representing recent recombination, with Gubbins (Croucher et al. 2015) using five iterations. Because Gubbins is known to work best for closely related isolates, we ran the tool only for the ST2 clade, which was found to be the largest recent clade in our collection. We then extracted the recombined regions and identified the genes that occurred in them. We searched the NCBI non-redundant nucleotide database using BLAST with the extracted recombinant sequences to identify the likely donor species of the recombined DNA. We also compared the results from Gubbins with those from ClonalFrameML (Didelot and Wilson 2015), which calculates Maximum Likelihood estimates at every site to identify recombinant fragments. For this programme, we used the relative transition versus tranversion rate of two and utilized the default priors for the gamma distribution in the program.

After removing the recombinant regions and reconstructing the phylogenetic tree, the root-to-tip distance was computed and plotted against the isolation year to assess the temporal signal. We conducted 1,000 bootstrap tests to evaluate the significance of the *R*-squared value, which was found to be greater than 80% of *R*-squared values from the bootstraps for the ST2 clade.

Subsequently we analyzed the data with BEAST v1.7 (Drummond and Rambaut 2007), testing various models that included a constant size population with strict molecular clock (with a log-normal distribution for base frequencies) and a GTR model with a gamma correction for among site variation. We then performed three independent chains for 50 million generations with sampling every ten generations. To control the convergence, we used the Effective Sample Size (ESS) values, defining convergence as ESS >200, after excluding 10 million initial states as burn-in. We found that the strict clock model always converged and therefore we based our results on the estimates from this model in this study. We used the TreeAnnotator tool available in the BEAST package to obtain the Bayesian tree and confidence intervals (CIs) for node ages.

## Results

**Fig. 1.—:**
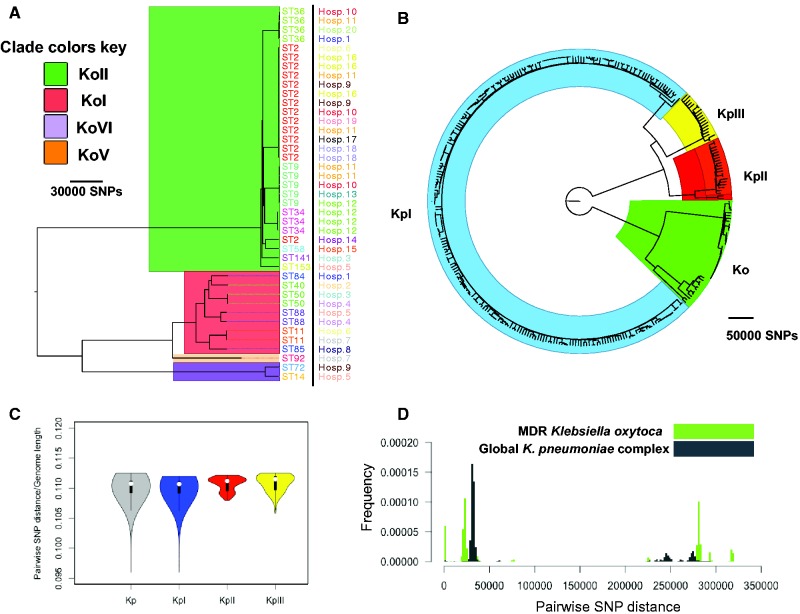
(*A*) Maximum Likelihood phylogenetic tree for the MDR *Klebsiella oxytoca* collection. Hosp. and ST stand for hospital of isolation and sequence type, respectively. The background colours signify the major phylogroups of *K. oxytoca*. (*B*) Combined phylogenetic tree of the global *Klebsiella pneumoniae* complex (Kp) collection and the MDR *K. oxytoca* (Ko) collection. The background colours correspond to the species within the global *K. pneumoniae* complex collection. KpI, KpII and KpIII correspond to *K. pneumoniae*, *Klebsiella quasipneumoniae*, and *Klebsiella variicola*, respectively. (*C*) Distribution of SNP distances for pairs of *K. oxytoca* and the whole *K. pneumoniae* complex (Kp) and each species within the *K. pneumoniae* complex*.* The values have been divided by the genome length of reference genome used for mapping. The boxes give the interquartile range and the whiskers indicate the boundary of 1.5 times the interquartile range. The white marker shows the median of the data and the coloured area is the probability density of the data at different values. (*D*) Distributions of pairwise SNP distances for the *K. oxytoca* collection and the global *K. pneumoniae* complex.

We first investigated the population structure of the MDR *K. oxytoca* isolates based on whole genome diversity. The population of *K. oxytoca* appears to consist of two major clades, both of which contain distinct sub-clades. Some of these sub-clades appear to have expanded recently within the studied population, suggesting recent dissemination of the pathogen (fig. 1*A*). It is known that the variants of the chromosomally encoded beta-lactamase-OXY define major phylogroups of this species, and the evolution of *bla*_oxy_ was thought to have occurred congruently with the evolution of *K. oxytoca* (Fevre et al. 2005). In line with this, we found that four variants of *bla*_oxy_ (*bla*_OXY1_, *bla*_OXY2_, *bla*_OXY6_, and *bla*_OXY5_) are present in the isolates and correspond to the phylogenetic groups KoI, KoII, KoVI, and KoV on the whole genome tree, respectively (fig. 1*A*). A comparison between phylogenetic trees for the core genome and the *bla*_oxy_ gene demonstrates that the trees are congruent for the major phylogroups (see supplementary fig. S1*A*, Supplementary Material online). However, within the KoII clade the *bla*_oxy_ tree shows some incongruency with the core genome. This within phylogroup incongruency may be due to recent recombination, and is also apparent for other core genes like *rpoB* and *dnaA* (see supplementary fig. S1*B* and *C*, Supplementary Material online), demonstrating that *bla*_oxy_ behaves like a true core gene. The majority of isolates are from KoII, and form one of the major clades, followed by KoI, which along with KoVI and KoV form the other major clade. KoV seems to be a closely related sister-group to KoI (fig. 1*A*). Our findings confirm that *bla*_oxy_ has a single common origin within *K. oxytoca*, and underwent vertical evolution within the major phylogenetic groups, as previously proposed (Fevre et al. 2005).

Previously published genomes of clinical *K. oxytoca* were from isolates pre-dominantly recovered from hospitals in the United States and therefore do not represent the global diversity of *K. oxytoca*. However, the phylogenetic distribution of the MDR *K. oxytoca* from our collection and the other available genomes indicates that the previously published genomes are interspersed with the UK&I isolates across the phylogenetic tree and within every major phylogroup. Indeed, *K. oxytoca* isolates collected from a single hospital’s intensive care units are spread across KoII, KoVI, KoI, and KoV (Roach et al. 2015; see supplementary fig. S2*A*, Supplementary Material online). Moreover, the genomes of two non-clinical isolates recovered from China are mixed with KoVI MDR isolates (Bao et al. 2013; Chen et al. 2013), and one environmental isolate from South Korea (Shin et al. 2012), one isolate recovered from panda faeces in China (Jiang et al. 2014), and one from a soap product in Germany (Hammerl et al. 2015) all appear to be closely related to MDR isolates in the KoI clade (see supplementary fig. S2*A*, Supplementary Material online). Some of the inter-country relationships that we identified in the KoI clade appear to share most recent common ancestors within the past few decades (see supplementary fig. S2*B*, Supplementary Material online), suggesting a global circulation of *K. oxytoca*. These results indicate that the MDR *K. oxytoca* within the collection from the UK&I are both highly diverse and linked to isolates of widely different origins and isolation sites. In addition, we found evidence that clinical isolates of *K. oxytoca* can be closely related to non-clinical isolates, suggesting the environment can serve as a potential reservoir for *K. oxytoca*.

To compare the population diversity of our collection with that of *K. pneumoniae* as the most closely related pathogen (Brisse and Verhoef 2001), we placed our collection in the context of a global *K. pneumoniae* complex population, which was dominated by *K. pneumoniae* (Holt et al. 2015; fig. 1*B*). A phylogenetic tree constructed from the two collections confirmed the complete separation between the species. The MDR *K. oxytoca* isolates appeared to be equally distant to each of the sub species of the *K. pneumoniae* complex, with a SNP distance/genome length of around 0.1 for the *K. oxytoca* genomes to each of the species of *K. pneumoniae* complex within the global collection (fig. 1*C*). In line with this, the ANI values between the two most closely and distantly related isolates from the MDR *K. oxytoca* and the global *K. pneumoniae* complex collection were 83.43% and 83.53%, respectively. Moreover, we found that MDR *K. oxytoca* was more diverse than *K. pneumoniae* complex species KpI (*K. pneumoniae*), KpII (*K. quasipneumoniae*) and KpIII (*K. variicola*) and the whole global *K. pneumoniae* complex collection, even though the MDR *K. oxytoca* collection was limited to clinical facilities in the UK and Ireland (fig. 1*D*). The ANI values for the most distantly related isolates seemed to be smaller for the MDR *K. oxytoca* (90.97%) than for the whole *K. pneumoniae* complex and each species within the collection (99.18%, 98.96%, 98.93%, and 92.87% for KpI, KpII, KpIII, and the whole *K. pneumoniae* complex populations, respectively). The low ANI value across the breadth of *K. oxytoca* isolates suggests that, similar to the global *K. pneumoniae* complex collection, which was split into three distinct species corresponding to the major phylogroups, defined by the major clades, the *K. oxytoca* species may need to be redefined and split into multiple species, defined by the major clades that we have identified here.

**Fig. 2.—:**
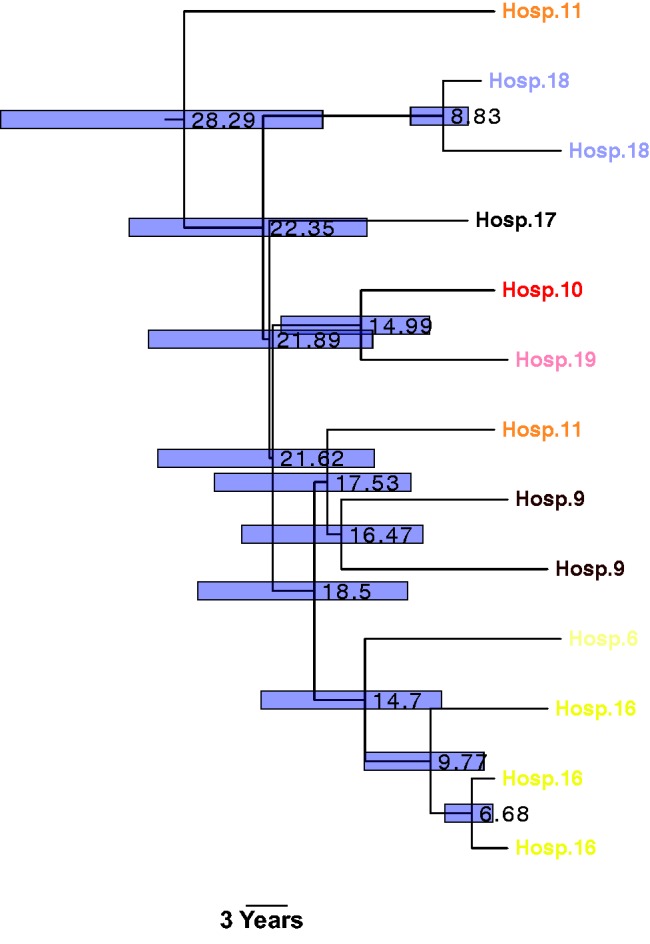
Dated Bayesian phylogenetic tree for the ST2 clade, generated using BEAST. The numbers on the nodes show node ages. The bars show the 95% confidence intervals.

In line with our finding of high diversity within *K. oxytoca*, we found that the collection was composed of 16 STs, of which only ST2, ST36, ST34, ST88, ST9, and ST50 were represented by >2 isolates. ST2 and ST9 are known to belong to a globally expanding beta-lactam resistant clonal complex (CC)2, implying again that our collection is part of a globally circulating *K. oxytoca* population (Izdebski et al. 2015). The clade representing ST2 appears to have undergone recent expansion in our collection (figs. 1*A* and 2). The temporal signal within ST2 allowed us to determine the nucleotide substitution rate to be around 2.5 SNPs per genome per year [95% CI: 0.87–4.89] (4.18×10^−^^7^SNPs/site/year), and thereby calculate divergence times for the isolates in the ST2 clade (fig. 2). The date of the most recent common ancestor was found to be around 30 years ago, and the dissemination of this ST within and across hospitals has therefore taken place since this date (fig. 2). Comparing the recombination rate (0.04 per genome per year) with the substitution rate for ST2, we can conclude that recombination has played a relatively minor role in shaping recent diversity of *K. oxytoca*. Recent recombination in *K. oxytoca* was found to generally involve the deletion or insertion of small genomic regions, with an average size of 11.79kb, but which could be up to 41kb (0.6% of the whole genome; see supplementary table S2, Supplementary Material online). Most recombination appears to occur in regions encoding bacteriophage genes, along with genes involved in conjugal transfer (see supplementary table S2, Supplementary Material online). In addition, genes involved in the SOS response such as the Lex transcriptional repressor and DNA polymerase subunit V, and also mercury resistance proteins, membrane proteins and fimbrial proteins were affected by recent recombination events. The recombination of membrane and fimbrial proteins indicates diversifying selection on surface structures, suggestive of selection due to interaction with the host immune system.

**Fig. 3.—:**
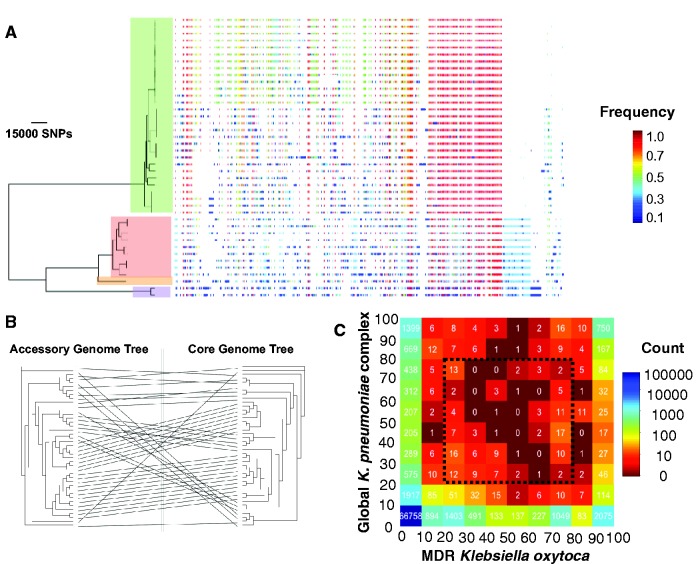
(*A*) Maximum Likelihood phylogenetic tree drawn for the core genome and the presence and absence of accessory genes in the accessory genome. The colours correspond to the frequency of accessory genes across the whole population. The clade colours are the same as in figure 1*A* and show *Klebsiella oxytoca* phylogroups. (*B*) Comparison between the core genome tree and the tree based on the presence-absence patterns of elements in the accessory genome. (*C*) The table of genes in the pan-genomes of *K. oxytoca* and *Klebsiella pneumoniae* complex. The colours signify the absolute frequency. The number on the axes denotes the relative frequency of the genes in either population (expressed in %). Note the first column and row of the table also shows genes that are completely absent in the other species. The dotted box shows acquired accessory genes in >20% and <80% of isolates within either species.

The genetic diversity of *K. oxytoca* is also reflected in the size of the accessory genome. The pan-genome of *K. oxytoca* contained 17,729 genes, of which 2,951 genes were in the core genome (in >99% of isolates), 376 genes were in the soft core genome (in >95% and <99% of isolates), 4,166 genes were in the shell genome (in >15% and <95% isolates) and 10,236 genes were in the cloud genome (in <15% of isolates; fig. 3*A*). This large accessory genome is likely to provide *K. oxytoca* with the genetic repertoire required for adaptation to multiple habitats. The pattern of the presence of accessory genes demonstrated a high level of concordance between the core genome tree and a tree built from the presence/absence of accessory genes (*P*-value for the association by Mantel test <0.001; fig. 3*B*). This suggests that each major clade has a unique set of accessory genes, which could be generated through early establishment of the accessory genome, or by the dominant mode of gene exchange being within clades (fig. 3*A* and *B*). Either would suggest that the major clades may have specific niche adaptations. In line with the distant phylogenetic relationship between the global *Klesbiella* collection and *K. oxytoca*, the two populations appeared to have a large set of core genes (1,399 genes for the global *K. pneumoniae* complex population and 2,075 genes for *K. oxytoca*), which were almost absent (in <10% of the population) in the other population (fig. 3*C*). Furthermore, the two populations harbored 66,857 accessory genes that were present in <10% of both collections. Together, these reflect the very distinct evolutionary forces that have shaped the genomic diversity of each species, and the likely distinct niches they occupy. However, figure 3*C* also shows that *K. oxytoca* and the *K. pneumoniae* complex species in the global collection do share a number of accessory genes that are variably present in both populations, indicating some shared adaptation to common niches. The list of shared accessory genes within the dotted box in figure 2*C*, which are present in 20–80% of the population of either collection, is provided in supplementary table S3, Supplementary Material online. Within this set of shared accessory genes, besides phage-related genes, are genes involved in virulence and drug resistance such as copper resistance proteins, various multidrug transporters, efflux pumps and iron transport proteins, suggesting that these genes may confer an adaptive advantage in both populations.

The sharing of gene pools between *K. oxytoca* and the *K. pneumoniae* complex population is reflected in the distribution of some conjugative multidrug resistance plasmids that also occur in the global *K. pneumoniae* complex population, some of which (such as Kpn3) are known to carry antimicrobial resistance genes (Pitout et al. 1998; see supplementary fig. S3*A*, Supplementary Material online). Moreover, these plasmids tend to occur more frequently in the nosocomially-acquired than in the community-acquired *K. pneumoniae* complex, which is suggestive of the spread of similar multidrug resistance plasmids in hospital settings between the two species (see supplementary fig. S3*B*, Supplementary Material online). The distribution of *K. pneumoniae* virulence factors also showed virulence factors that were widespread across the MDR *K. oxytoca* population and may be present in strains from all clades (see supplementary fig. S4, Supplementary Material online). These *K. pneumoniae* virulence genes include the *fyuA* and *ybtAPTQ* genes of the yersiniabactin gene cluster, involved in iron acquisition (Lawlor et al. 2007), the *ybbW* and *allBCRAS* genes of the allantoinase gene cluster, involved in the metabolism of allatonin (Chou et al. 2004), and the *mrkAB* genes that are involved in the production of mannose-resistant fimbriae and biofilm formation (Jagnow and Clegg 2003). While our results indicate sharing with *K. oxytoca* of genes known to be involved in virulence of *K. pneumoniae*, whether these genes are actually involved in virulence in *K. oxytoca* will need evidence from functional studies.

**Fig. 4.—:**
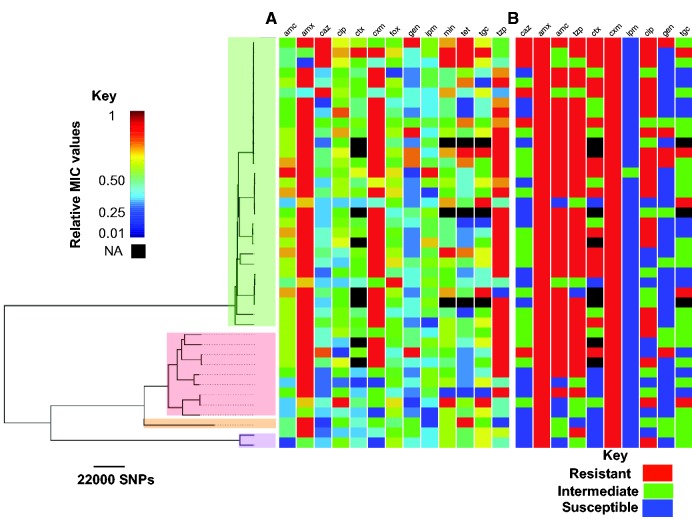
(*A*) Distribution of MICs and (*B*) resistance phenotype across the phylogenetic tree. The clade colours are the same as in figure 1*A* and show *K. oxytoca* phylogroups. Abbreviations of the antibiotics are: amoxicillin (amx), cefuroxime (cxm), amoxicillin–clavulanate (amc), cefotaxime (ctx), cefoxitin (fox), imipenem (ipm), piperacillin–tazobactam (tzp), ciprofloxacin (cip), ceftazidime (caz), gentamicin (gen), tigecycline (tgc), minocycline (min) and tetracycline (tet). We normalized the MICs for each antibiotic such that the maximum and minimum MICs take values 1 and 0, respectively. Note that we show the phenotypes only for antibiotics for which clinical breakpoints were defined.

**Fig. 5.—:**
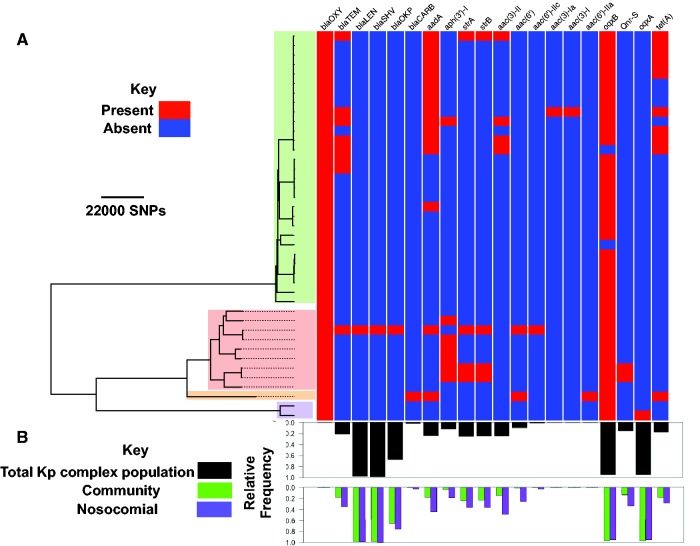
(*A*) Distribution of known antimicrobial resistance genes across the phylogenetic tree. The clade colours are the same as in figure 1*A* and show *Klebsiella oxytoca* phylogroups. (*B*) Relative frequencies of plasmid replicons, found in MDR *K. oxytoca* genomes, in the global *Klebsiella pneumoniae* population (black barplot) and its nosocomial and community sub-populations (grouped green and purple barplots), for each resistance gene.

*Klebsiella oxytoca* is known to have a similar antimicrobial resistance profile to *K. pneumoniae*. Despite the variation in the MICs for every antimicrobial (fig. 4*A* and see supplementary fig. S5, Supplementary Material online), at the phenotypic level, there were different degrees of variation: our isolates were extensively resistant to amoxicillin (S:0, I:0, R:41), cefuroxime (S:0, I:0, R:41) and piperacillin–tazobactam (S:3, I:7, R:31). In contrast, resistance was less common for amoxicillin–clavulanate (S:11, I:0, R:30; known to inhibit chromosomally encoded beta-lactamases; Arakawa et al. 1989), ciprofloxacin (S:9, I:10, R:22) and ceftazidime (S:26, I:9, R:6). Our isolates were extensively susceptible to imipenem (S:41, I:0, R:0), gentamicin (S:33, I:3, R:5), and tigecycline (S:20, I:13, R:5; fig. 4*A* and *B*). As expected, we observed a strong correlation between MIC values for some antimicrobials, most notably antimicrobials with similar resistance mechanisms (see supplementary fig. S6, Supplementary Material online). For every antimicrobial where we analyzed the relationships between elevated resistance level (MICs) and the genotype, if there was variation in resistance phenotype, then these were spread across the phylogenetic tree, implying that mechanisms increasing resistance may be acquired independently by sub-clades (fig. 4*A*). However, the MICs for beta-lactams seemed to be higher for the KoII group than for the KoI group (see supplementary fig. S7, Supplementary Material online). This difference might be due to expression levels rather than the enzyme type (Gheorghiu et al. 1997; Livermore 1995) because besides *bla*_OXY_, which was not linked with resistance in our dataset, only one isolate seemed to have gained *bla*_SHV_ (an ESBL) and *bla*_LEN_, which are specific to *K. pneumoniae*, and nine isolates had *bla*_TEM_ (fig. 5*A*). For the majority of other antimicrobial resistance genes, the genes are also found in the global *K. pneumoniae* complex population and they occurred more frequently in the nosocomial sub-population than the community sub-populations of the *K. pneumoniae* complex (fig. 5*B*). The low frequency of known ESBLs suggests that beta-lactam resistance may be driven by other mechanisms that alter the expression level or the structure of existing beta-lactamases in *K. oxytoca*, rather than the acquisition of novel beta-lactamases. This potentially includes occurrence of chromosomal mutations or different expression levels of penicillin-binding protein AmpH, which is present in every isolate.

The pattern of acquisition of other known antibiotic resistance determinants indicates that antimicrobial resistance determinants have been acquired independently across the population and their presence is linked with elevated MICs for some clades (fig. 5*A*). Increased tetracycline resistance was observed in sub-clades of ST2 and was associated with the presence of copies of tetracycline efflux *tetA* genes (figs. 4*A* and 5*A*). In addition, gentamicin resistance genes, in particular *strA* and *strB* phosphotransferase genes have been acquired in a few isolates throughout the phylogenetic tree and exhibit strong association with elevated MICs for gentamicin and gentamicin resistant phenotype, showing enzymatic resistance is responsible for gentamicin resistance in *K. oxytoca* (fig. 5*A*;*P*-value < 0.01 obtained from Student’s *t*-test for the significance of association between gene presence/absence variable (predictor variable) and MICs (dependent variable) in a linear regression model). Besides the efflux pump-encoding *oqxB* gene, present in almost every isolate and the *oqxA* gene present only in isolates in the KoVI clade, two KoI isolates appear to have the quinolone resistance gene *qnrS*, but none of these genes seem to be associated with variations in the MICs for ciprofloxacin. In contrast the non-synonymous mutations at the known codon position 83 of DNA gyrase A (Gruger et al. 2004), which is present in 95% of resistant strains and absent in 90% of susceptible/intermediate strains, appear to primarily account for ciprofloxacin resistance. Furthermore, specific non-synonymous mutations at position D87 of DNA gyrase A and at position E461 of DNA topoisomerase IV subunit B also occurred in ciprofloxacin resistant isolates. Given the decreased susceptibility to ciprofloxacin that has been reported across Europe (Brisse et al. 2000), the multiple independent emergence and spread of resistance determinants could greatly limit the use of ciprofloxacin to treat *K. oxytoca.*

## Discussion

In this study we used whole genome sequencing to elucidate the population structure of multidrug resistant *K. oxytoca*, as the second most clinically prevalent species of the *Klebsiella* genus (Broberg et al. 2014). Our results show that the MDR *K. oxytoca* population is highly diverse and that some clades have undergone recent expansion in our population. Despite the high level of divergence between *K. oxytoca* and the *K. pneumoniae* complex, similar virulence and antimicrobial resistance mechanisms appear to operate within either species.

Our results provide evidence for the existence of a shared gene pool and the role of convergent evolution in developing similar virulence and, to a lesser degree, antimicrobial resistance mechanisms in these two closely related species, presumably in the face of similar ecological challenges. This suggests that utilizing a shared gene pool via horizontal gene transfer may facilitate adaptation to new environments across these species concurrently, as has been proposed in other bacterial species (Cooper 2007; Wiedenbeck and Cohan 2011), which in turn suggests that these organisms may occupy converging niches. Understanding whether or not these mechanisms originate from the ancestral state (parallelism) or have been acquired from unrelated sources (Stern 2013) will require a broader sampling from other *Klebsiella* species and also from more closely related intermediate species between *K. oxytoca* and *K. pneumoniae*. In doing so we will be able to better understand the evolutionary forces that delineate *Klebsiella* species, in particular in connection with specific and similar habitats that each species occupies.

As an opportunistic pathogen, *K. oxytoca* is known to have environmental sources and therefore the environment may serve as a potential reservoir for MDR *K. oxytoca*. This not only includes contaminated hospital equipment, but also the carriage of the pathogen by healthy people between distant geographical regions and subsequent introduction into hospitals (Decre et al. 2004; Lowe et al. 2012). Our observation of the rapid dissemination of one *K. oxytoca* clone across what are geographically distant hospitals suggests that transmission may not be directly between hospitals, but may involve carriage in the general population. This would suggest that MDR strains have already spread across the country and on some occasions will give rise to infections within hospital settings. This epidemiological pattern is what would be expected for a commensal organism and makes outbreak control more difficult than for true nosocomial pathogens.

In this study we have used whole genome sequencing to conduct a comparative genomic analysis for two closely related opportunistic pathogens. Compared with *K. pneumoniae*, *K. oxytoca* has been less well studied and this study has provided the very first insights into the population diversity of *K. oxytoca*. To understand the broader patterns of *K. oxytoca* epidemiology and evolution; however, a collection from a wider geographical area that also contains drug susceptible strains will be required. This will serve as the basis to understand the population structure of *K. oxytoca* and elucidate the global epidemiology of *K. oxytoca*.

## Supplementary Material

Supplementary data are available at *Genome Biology and Evolution* online.

## Supplementary Material

Supplementary Data
