# Supplementary material for: Population Structure of Multidrug-Resistant Klebsiella oxytoca within Hospitals across the United Kingdom and Ireland Identifies Sharing of Virulence and Resistance Genes with K. pneumoniae
Source: Genome Biol Evol. 2017 Mar 1;9(3):574–84. doi: 10.1093/gbe/evx019 (PMC5381567; doi:10.1093/gbe/evx019)
Supplement: Supplementary Data [file evx019_Supp.zip › SupplementaryFigures.pdf]

Supplementary Figures

Population structure of multidrug resistant *Klebsiella oxytoca* within hospitals across the UK and Ireland identifies sharing of virulence and resistance genes with *K. pneumoniae*

Danesh Moradigaravand, Veronique Martin, Sharon J. Peacock and Julian Parkhill

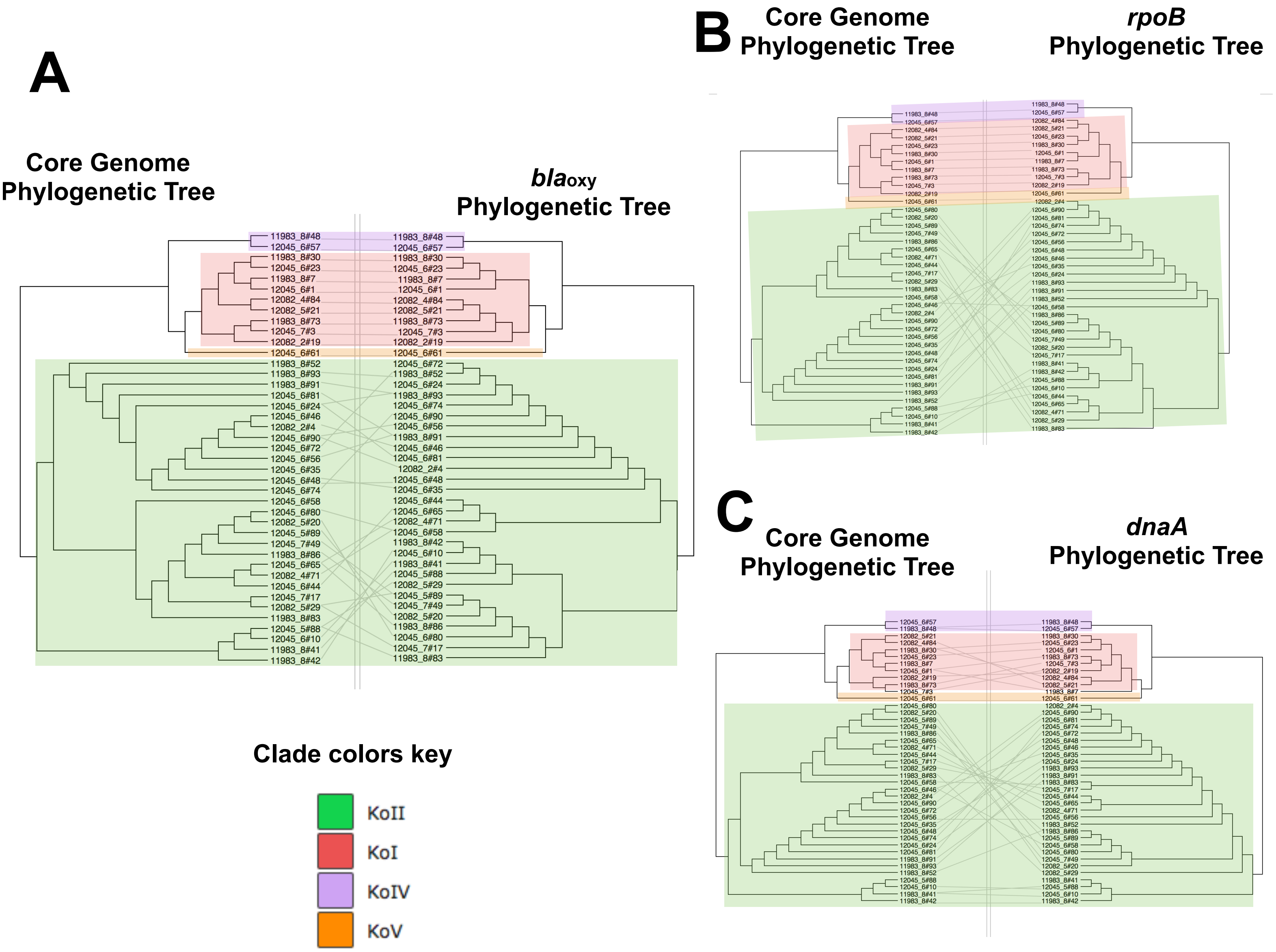

Figure S1 Comparison between the dendrograms generated for the core genome and the A) bla_{oxy}, B) *rpoB* and C) *dnaA* genes. The vertical lines connect identical tips on the trees. The background colors correspond to the major phylogroups.

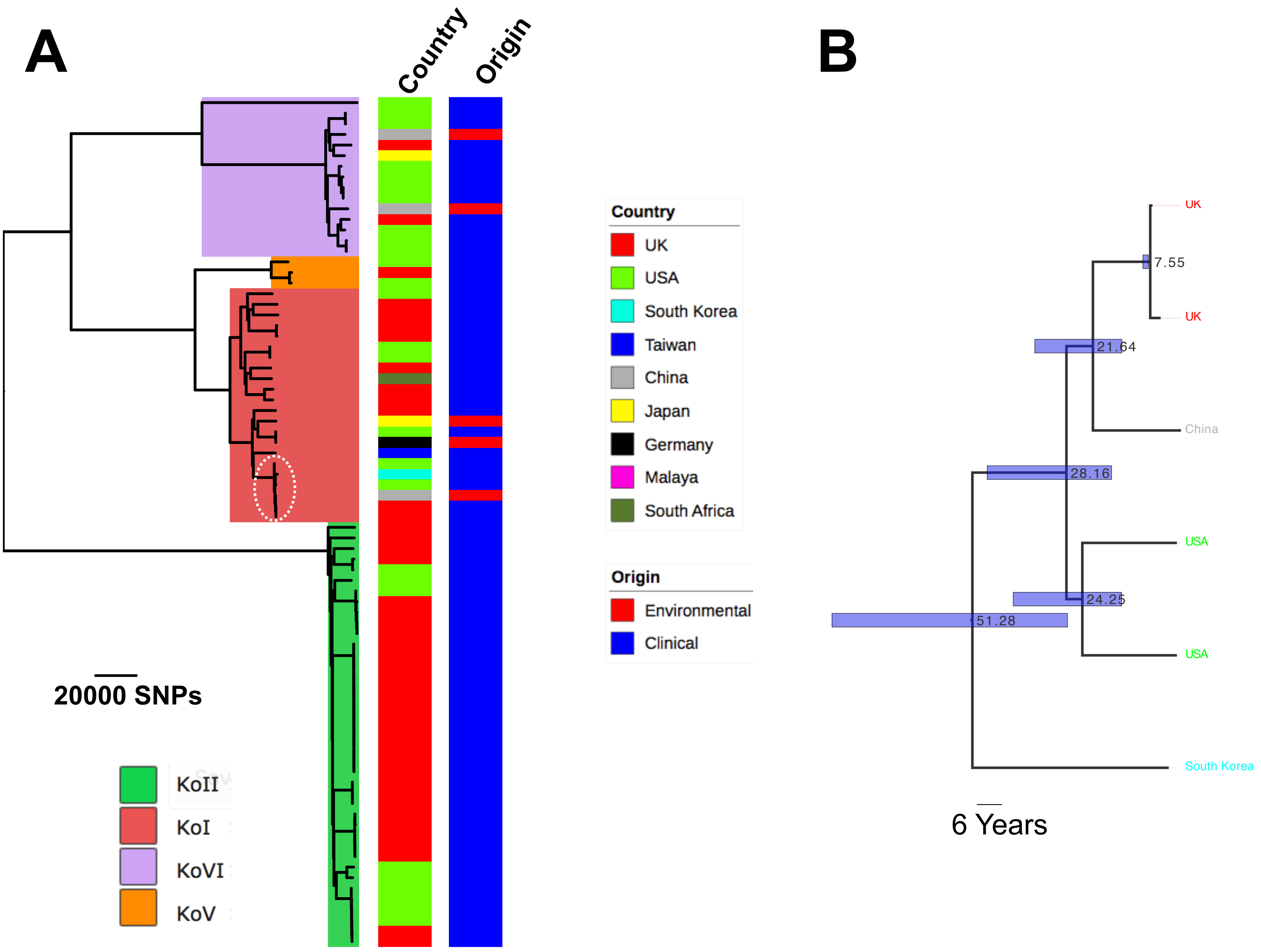

Figure S2 A) Maximum Likelihood phylogenetic tree for isolates in the MDR *K. oxytoca* (BSAC) and previously published *K. oxytoca* genomes. B) The sub-tree of the isolates in the dotted circle in the KoI phylogroup in A). The node numbers show the age of divergence and the bars show 95% confidence interval. The most recent isolate in the tree was recovered in 2014.

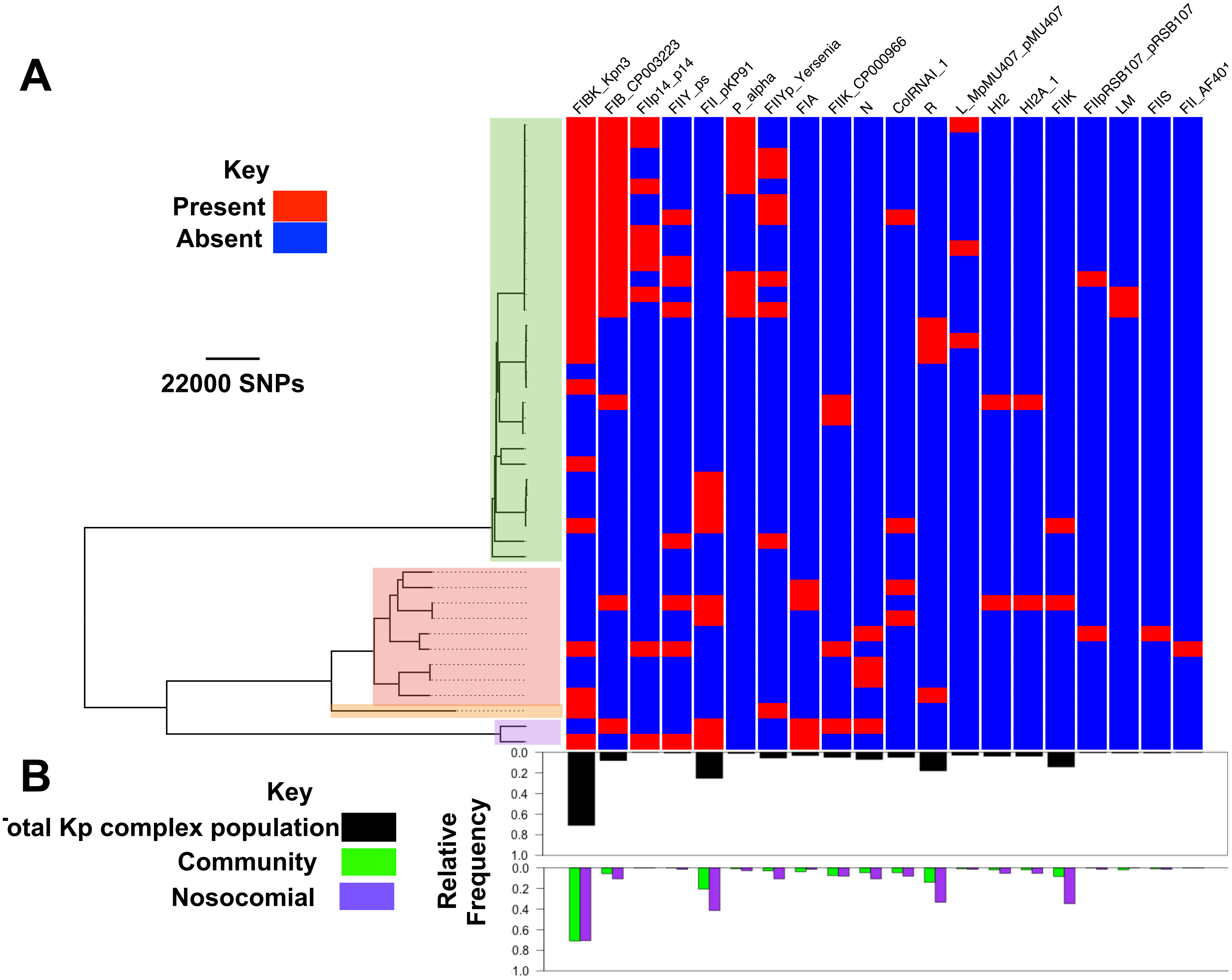

Figure S3 A) Distribution of plasmid replicons across the phylogenetic tree. The clade colours are the same as in Figure 1A and show *K. oxytoca* phylogroups. B) Relative frequencies of plasmid replicons, found in MDR *K. oxytoca* genomes, in the global *K. pneumoniae* population (black barplot) and its nosocomial and community sub-populations (grouped green and purple barplots), for each plasmid replicon. The background clade colors correspond to the major phylogroups, as defined in Figure S1.

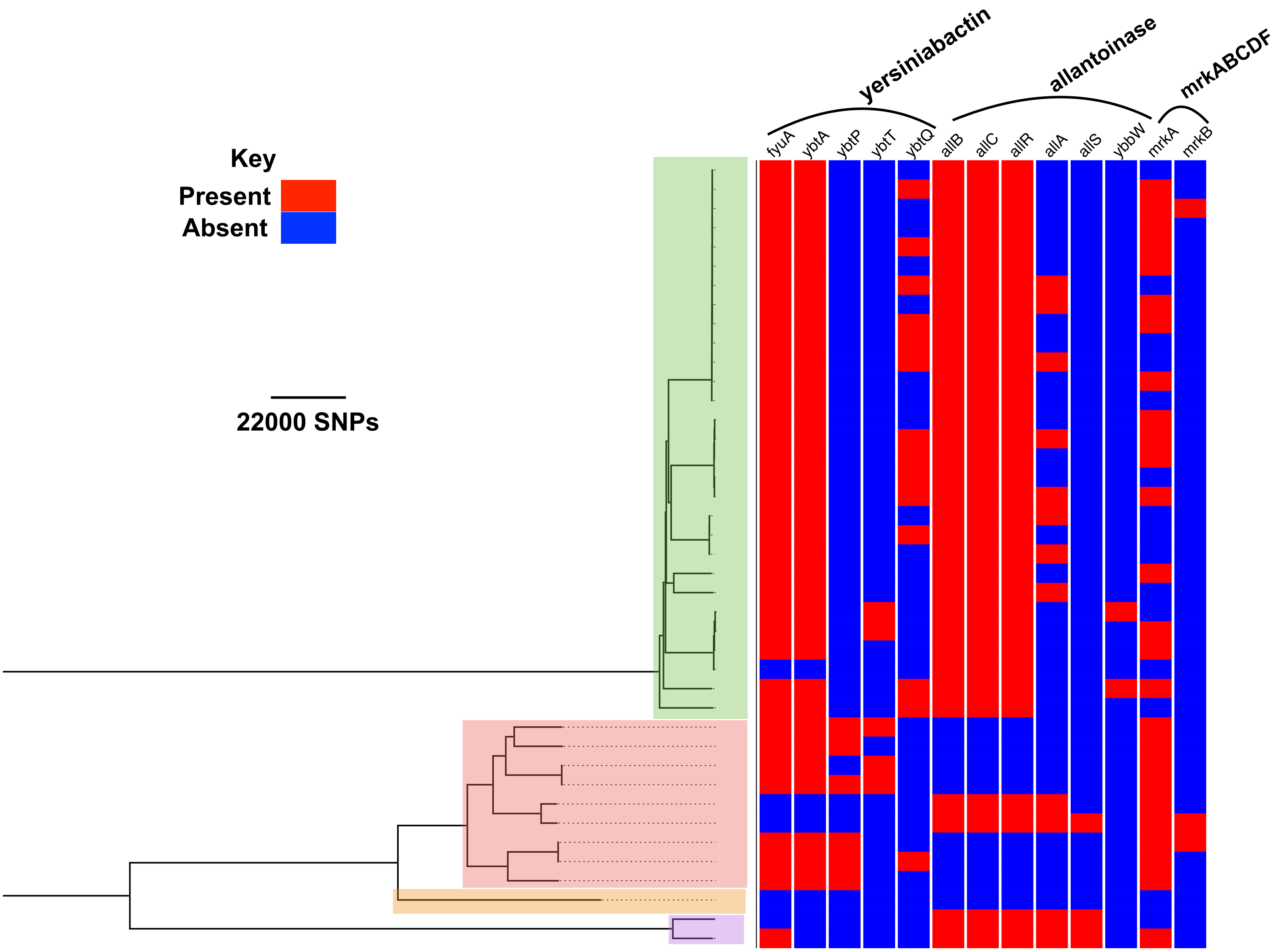

Figure S4 Distribution of *K. pneumoniae* virulence factors across *K. oxytoca* genomes. The clade colours are the same as in Figure 1A and show *K. oxytoca* phylogroups. The background clade colors correspond to the major phylogroups, as defined in Figure S1.

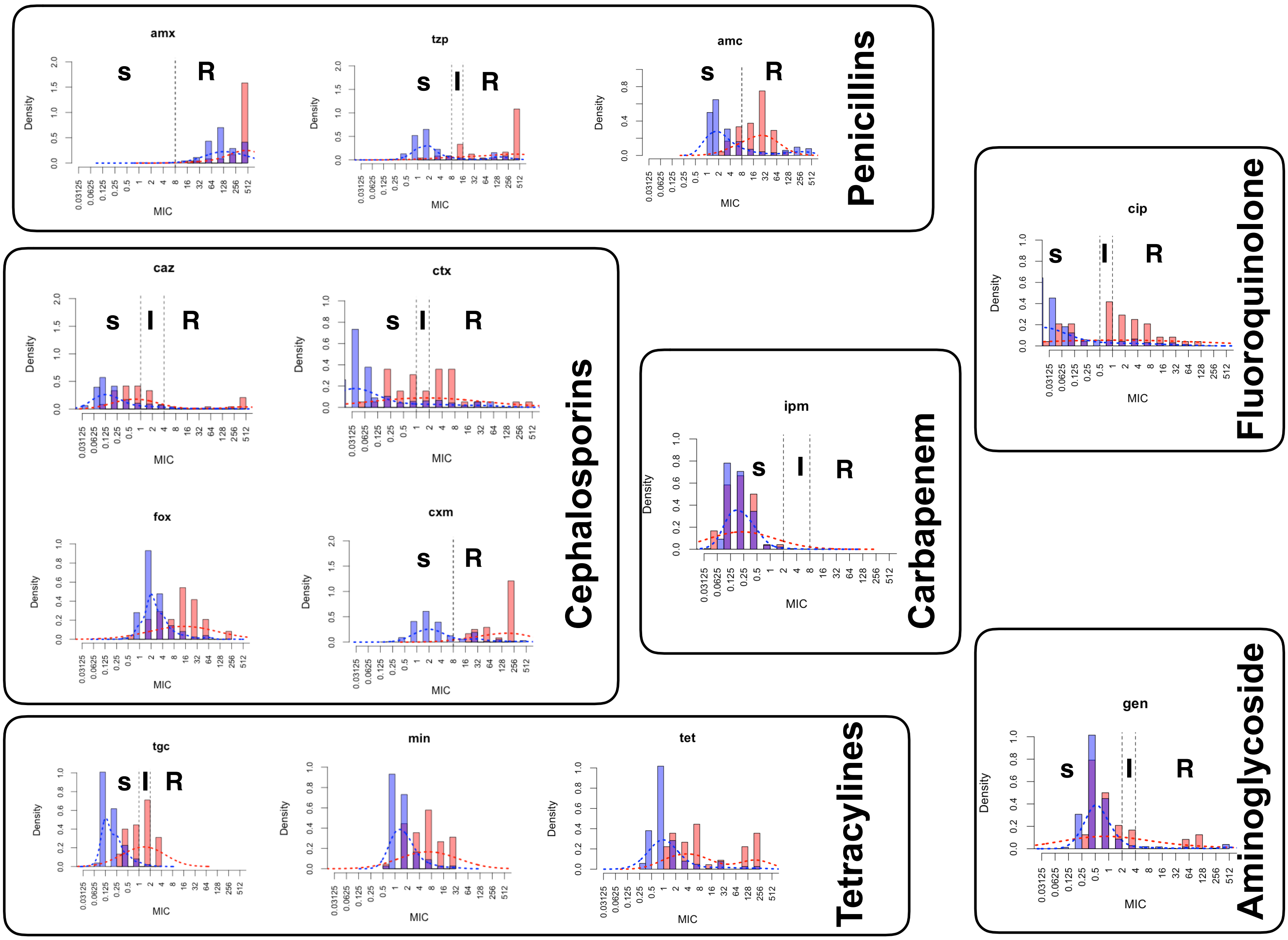

Figure S5 MIC distributions for various antimicrobials tested in this study (in red) and the distributions for the EUCAST collection. Abbreviations of the antibiotics are: amoxicillin (amx), cefuroxime (cxm), amoxicillin-clavulanate (amc), cefotaxime (ctx), cefoxitin (fox), imipenem (ipm), piperacillin-tazobactam (tzp), ciprofloxacin (cip), ceftazidime (caz), gentamicin (gen), tigecycline (tgc), minocycline (min) and tetracycline (tet). The “R“, “I“ and ”S” letters stand for resistant, intermediate and susceptible, respectively. The dashed red and blue curves show estimated continuous MIC distributions for the EUCAST and our isolates, respectively. No clinical breakpoint was defined for tetracycline (tet), minocycline (min) and cefoxitin (fox).

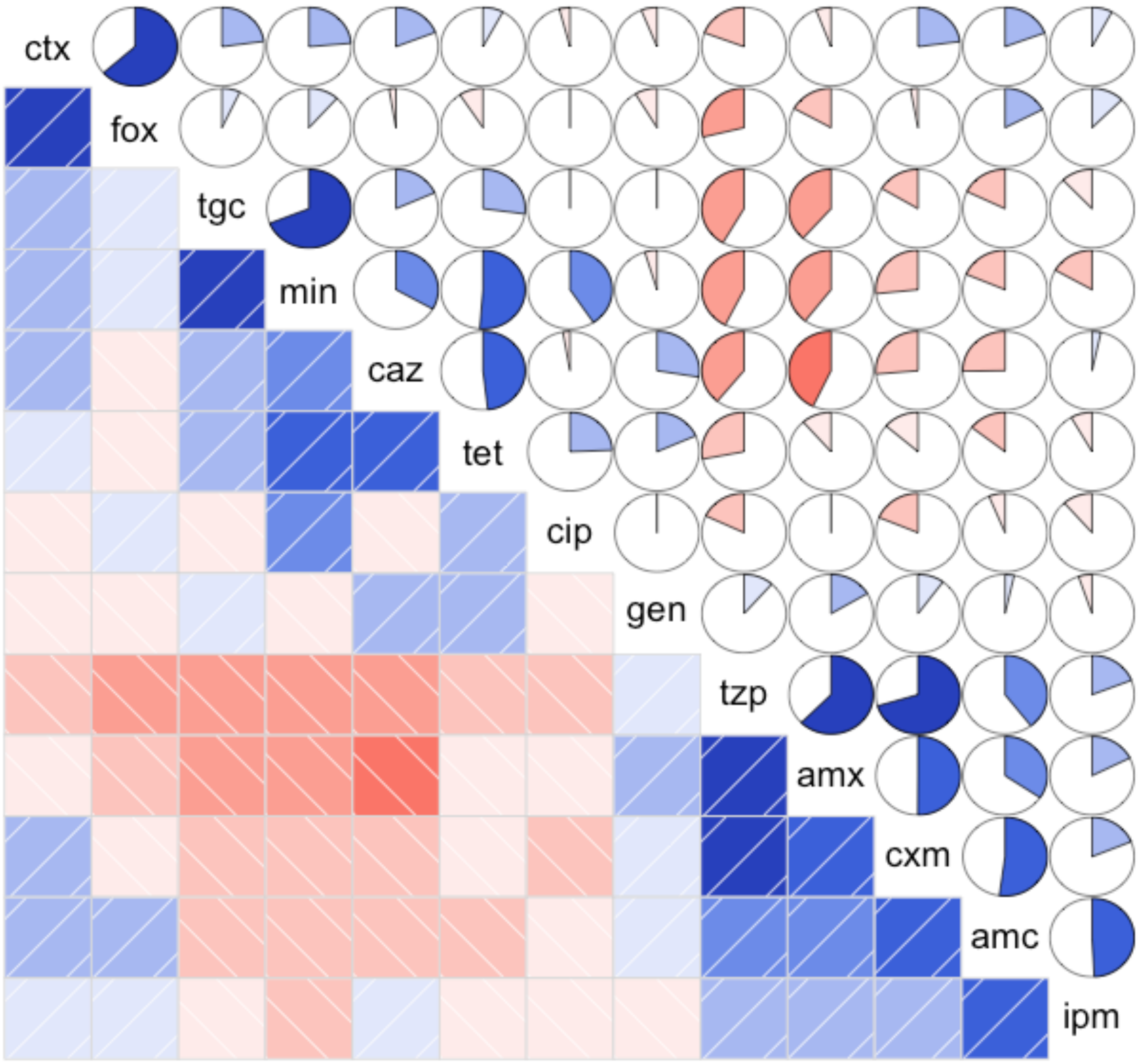

Figure S6 The correlogram of the MIC values for the antibiotics tested in this study. The red and blue colours signify negative and positive correlations, respectively. Abbreviations of the antibiotics are: amoxicillin (amx), cefuroxime (cxm), amoxicillin-clavulanate (amc), cefotaxime (ctx), cefoxitin (fox), imipenem (ipm), piperacillin-tazobactam (tzp), ciprofloxacin (cip), ceftazidime (caz), gentamicin (gen), tigecycline (tgc), minocycline (min) and tetracycline (tet). The pie charts and the depth of the shading both indicate the magnitude of the correlation.

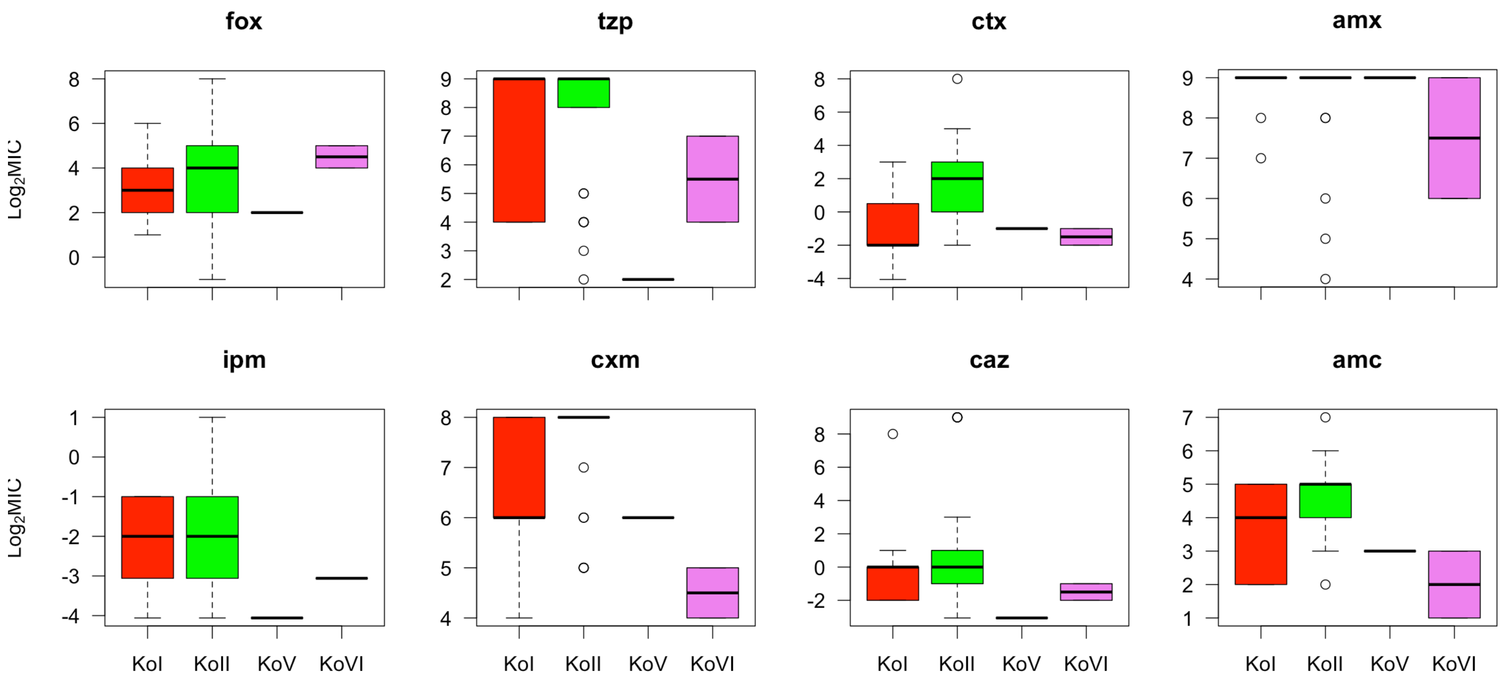

Figure S7 Distribution of log_2(MIC) values across the major phylogroups of *K. oxytoca*. The boxes give the interquartile range, the whiskers indicate the boundary of 1.5 times the interquartile range, and outliers are defined as the points beyond this range. Abbreviations of the antibiotics are: amoxicillin (amx), cefuroxime (cxm), amoxicillin-clavulanate (amc), cefotaxime (ctx), cefoxitin (fox), imipenem (ipm), piperacillin-tazobactam (tzp) and ceftazidime (caz).
